# Adverse occupational outcome among workers with occupational asthma: A systematic review and meta-analysis of influencing factors

**DOI:** 10.5271/sjweh.4282

**Published:** 2026-07-01

**Authors:** Lukas S Damerau, Matthias W Helm, Julia Pieter, Marcial Velasco Garrido, Volker Harth, Hanno Hoven, Alexandra M Preisser

**Affiliations:** 1 Institute for Occupational and Maritime Medicine (ZfAM), Medical Center Hamburg-Eppendorf (UKE), Hamburg, Germany.; 2 Department of Sociology and Political Science, Norwegian University of Science and Technology (NTNU), Trondheim, Norway.

**Keywords:** return to work, specific inhalation challenge, unemployment

## Abstract

**Objectives:**

Occupational asthma (OA) often interferes with workers’ ability to maintain employment. We synthesized the prevalence of adverse occupational outcome (AOO)—unemployment, chronic sick leave, disability, and early retirement—caused by OA.

**Methods:**

Following PRISMA methodological recommendations and PROSPERO registration (CRD42024528750), we searched PubMed, Scopus, and Web of Science for studies published between January 1980 and September 2024 that reported quantitative employment outcomes in adults with OA. Risk of bias was assessed using the Joanna Briggs Institute Checklist for Prevalence Studies. Pooled prevalences were calculated using random-effects generalized linear mixed models on the logit scale with Hartung–Knapp 95% confidence and prediction intervals (CI and PI).

**Results:**

A total of 25 studies comprising 3393 participants were included. The pooled prevalence of AOO was 35.9% (95% CI 28.6–43.9; I^2^=86.0%; 95% PI 10.2–73.4). Prevalence of AOO varied by: (i) data sources (registry/compensation: 50.1% versus clinic: 32.0%; P=0.015); (ii) study size (>80 participants: 43.4% versus ≤80: 27.9%; P=0.033); (iii) baseline forced expiratory volume in 1 second (FEV_1_) (≤85% predicted: 38.2% versus >85% predicted: 13.8%; P=0.008); and (iv) exposure duration before symptom onset (>7.1 years: 35.7% versus ≤7.1 years: 15.7%; P=0.015). Heterogeneity across studies was substantial and several subgroups included fewer than five studies.

**Conclusions:**

More than one-third of workers with OA experience an AOO. The findings highlight the need for harmonized outcome definitions and for interventions integrating early referral, exposure control, and structured return-to-work programs to avoid AOO.

Occupational asthma (OA) is one of the leading work-related respiratory diseases. Exposures at the workplace are estimated to account for approximately 16% of adult-onset asthma in the working-age population (1). Despite its public health relevance, OA remains underrecognized, particularly in high-risk sectors such as manufacturing and agriculture, where limited diagnostic resources and varying reporting practices hinder accurate estimates (2). Quantifying the disease burden requires precise case definitions, particularly given the evolution of diagnostic criteria over time (2, 3). OA is a distinct clinical entity within the broader spectrum of work-related asthma. While this classification encompasses both OA and work-exacerbated asthma, which is defined as the aggravation of pre-existing asthma by workplace conditions, OA is etiology-specific. It is characterized by variable airflow limitation or hyperresponsiveness caused by exposures attributable to a specific occupational environment. OA comprises two main phenotypes: sensitizer-induced asthma, which involves an immunological latency period, and irritant-induced asthma, which typically occurs without latency following high-level exposure (4). Although earlier research frequently focused on sensitizer-induced forms, irritant-induced asthma is increasingly recognized as a distinct phenotype that may be associated with a poorer long-term prognosis (5, 6).

The economic consequences of OA are substantial. In the United Kingdom alone, annual costs are estimated at £100 million, encompassing healthcare utilization and productivity losses (7). In low- and middle-income countries, underdiagnosis, limited access to specialist care, and uneven access to occupational-health systems can exacerbate the impact of OA on employment, even though reported population-attributable fractions are generally in the single to low-double digits (8).

Clinical management of OA typically prioritizes cessation of exposure to causative allergens. While this approach can improve respiratory outcomes, it may lead to adverse occupational outcomes (AOO) for the affected worker, including unemployment, chronic sick leave, disability, and early retirement. Previous studies report sizeable impacts. For example, one study using French data observed that 44% of workers with OA had left their previous job and 25% were unemployed at follow-up, while 46% of the total cohort experienced a reduction in income (9). Socioeconomic consequences can be particularly stark in low- and middle-income countries settings. A recent Brazilian cohort study reported catastrophic health expenditures among most patients (10). Alternatives that aim to reduce rather than cease exposure have shown mixed effectiveness for maintaining employment and controlling symptoms (11). Beyond clinical and economic differences, systemic factors also shape employment outcomes. Differences in employment protection, workers’ compensation, and return-to-work policies across countries may contribute to the observed variation in work-loss prevalence.

Previous studies vary in outcome definitions, lengths of follow-up, and settings. To the best of our knowledge, a comprehensive quantitative synthesis is lacking. To address this gap, we systematically reviewed studies from 1980–2024 to estimate the prevalence of AOO and to assess heterogeneity by region and study-level indicators, including diagnostic method, spirometry, exposure duration, and study setting.

## Methods

### Search strategy and study selection

This systematic review and meta-analysis followed PRISMA 2020 guidelines (supplementary material, www.sjweh.fi/article/4282) and was registered in PROSPERO (CRD42024528750). We searched PubMed/MEDLINE, Scopus, and Web of Science core collection for records published from January 1980 to September 2024. Search strategy combined medical subject heading (MeSH) terms and database-specific keywords related to OA and employment-related outcomes (supplementary table S1). Grey literature, trial registries, and reference lists were not searched. We included peer-reviewed randomized or observational studies that reported quantifiable AOO in adults with OA. We excluded pediatric populations, case reports, reviews, editorials, conference abstracts, non-human studies, and studies without extractable outcome data. In addition, studies reporting <20 OA cases were excluded to ensure sufficient precision for descriptive statistics and prevalence estimates. Two reviewers independently screened titles/abstracts and full texts in a multi-stage process, resolving discrepancies by consensus.

In finalizing the data extraction framework, we refined the outcome definition and appraisal method to ensure consistency across heterogeneous designs. The primary endpoint was specified as a binary employment status (“in regular paid work” versus “not in regular paid work”) to ensure uniform denominators as many studies did not distinguish between job change, reduction, or cessation. Because most included studies were single-arm observational reports in which employment outcomes were secondary, comparative analyses were not feasible. The Joanna Briggs Institute (JBI) Checklist for Prevalence Studies (12) was used to assess methodological quality instead of the initially planned NOS/ROBINS-I tools, reflecting the predominance of cross-sectional designs. These clarifications were made early in the review process and did not alter the scope or objectives of the review.

### Data extraction and quality assessment

Extracted data included study design, sample size, mean age, sex distribution, employment outcomes (unemployment, chronic sick leave, disability, early retirement), and potential determinants [baseline lung function: forced expiratory volume in 1 second (FEV_1_), forced vital capacity (FVC), exposure duration before symptom onset, duration of symptoms until diagnosis, and longitudinal change in FEV_1_ (ΔFEV_1_)] (supplementary extraction table).

Using the JBI checklist, for each study, we calculated a percentage score as “Yes”/(“Yes”+“No”+“Unclear”), excluding items marked “not applicable” from the denominator. Studies were assigned to one of three categories: high (≥80%), moderate (60–79%), or low (<60%) quality. These categories were used for subgroup analyses to explore heterogeneity.

### Adverse occupational outcome

AOO was defined as “not being in regular paid work at the relevant time of assessment”. In the main analysis, AOO was treated as a binary variable (in paid work versus not). Regular old-age retirement was not considered an AOO and excluded from the analysis. For descriptive and sensitivity analyses, we also examined the specific types of AOO reported in the studies. These included (i) unemployment, (ii) chronic sick leave, (iii) disability, and (iv) early retirement. Disability was defined as the formal receipt of a disability pension or a medical determination of permanent work incapacity due to OA, as specified in the individual studies. Each participant was counted in only one of these categories, according to how the original study described the outcome. Where studies used country-specific terms (eg, “invalidity pension”, “sick allowance”), these were assigned to the category that best matched their functional meaning. Employment changes without actual job loss (eg, job reassignment or reduction in working hours) were not considered AOO. In studies that allowed multiple answers for employment status (so that category totals exceeded 100%), only the overall “not in paid work” status was analyzed, and such studies were not used in the separate analyses of the specific outcome types (13–15). In cross-sectional studies, employment status was taken at the time of assessment. In longitudinal studies, the outcome was taken from the last available follow-up. Participants with unknown or missing employment status at the relevant time point were excluded from the prevalence calculations.

### Data transformation and harmonization

To ensure comparability across studies, validated transformation methods were applied when necessary. For studies reporting median and range instead of mean and standard deviation (SD), we estimated the latter using established formulas by Hozo et al (16), which account for sample size and distribution range. For datasets providing interquartile range, we employed sample-size-adjusted estimators proposed by Wan et al (17) to minimize approximation error. For studies reporting several OA subgroups, such as different exposure groups, we combined these groups to obtain overall counts and event numbers for AOO.

### Data synthesis and statistical analysis

We summarized study-level proportions using a generalized linear mixed model (GLMM) with random effects, applying a logit transformation with back-transformation to obtain pooled estimates with 95% confidence intervals (CI). Between-study variance (τ^2^) was estimated by maximum likelihood, and we used the Hartung-Knapp approach to obtain pooled 95% prediction intervals (PI). These intervals reflected the range in which the true prevalence of a comparable future study would be expected to fall, thereby illustrating the extent of between-study heterogeneity. Heterogeneity was further assessed using Cochran’s *Q* to test for its statistical significance and the *I*^2^ statistic to quantify the proportion of total variation attributable to between-study heterogeneity rather than sampling error. The primary endpoint was the prevalence of AOO, calculated as the number of participants with OA who were not in regular employment (unemployment, chronic sick leave, disability, or early retirement) divided by the total number of study participants with OA. Robustness was examined with leave-one-out influence analysis and Baujat plots. Potential small-study effects were assessed using Egger’s regression together with contour-enhanced funnel plots and Duval and Tweedie’s trim and fill method. We additionally applied limit meta-analysis, an approach that adjusts the pooled effect for small-study bias via regression-based extrapolation to zero standard error. Sensitivity analyses included disaggregating the composite endpoint into its four components via separate GLMM. We also explored study-level and clinical/demographic factors (eg, region, study design, study size, year, age, sex, lung function, exposure duration, setting, diagnostic method, study quality) as potential subgroups. Given the observed lower heterogeneity within the UK subgroup compared to the global dataset, we performed sensitivity analyses for the UK subgroup by excluding national registry data (14). Data-driven cut points (eg, median splits) were applied for continuous variables. Owing to substantial missingness in key variables, no meta-regression was undertaken. Exploratory stratified summaries are provided as descriptive, non-inferential analyses. All calculations were conducted in R (version 4.5.1) using the meta, metafor, and metasens packages (18).

## Results

### Study selection

**Figure 1:**
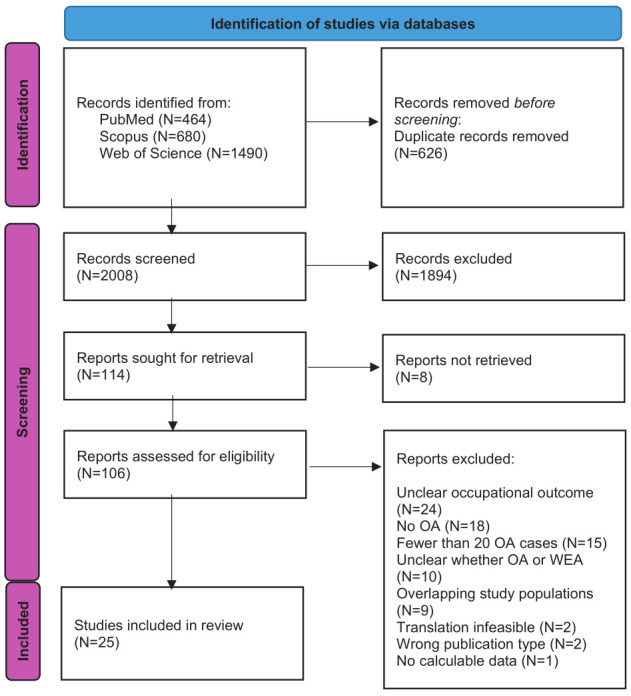
Flowchart of the study selection process (PRISMA). OA=occupational asthma; WEA=work-exacerbated asthma.

Figure 1 summarizes the selection process. The searches in PubMed, Scopus, and Web of Science identified 464, 680, and 1490 records, respectively. After removing 626 duplicates, 2008 titles/abstracts were screened, of which 1881 were excluded. We attempted to obtain 114 full reports of which 8 could not be retrieved. In total, 106 full-text publications were assessed for eligibility. Reasons for exclusion (supplementary table S2) included unclear occupational outcome (*k*=24 studies), no OA (*k*=18), <20 OA cases (*k*=15), unclear whether OA or work-exacerbated asthma (*k*=10), overlapping study populations (*k*=9), translation infeasible (*k*=2), wrong publication type (*k*=2), and no extractable quantitative data (*k*=1).

### Characteristics of included studies

Table 1 presents the 25 included studies (6, 9, 10, 13–15, 19–37) (total N=3393 participants) published between 1982 and 2024. Sample sizes were 25–723 (median 80), and mean ages of participants 35.5–58.7 years. Of these, 9 were cross-sectional studies and 16 longitudinal (follow-up 0.5–10.6 years). Study quality was rated as high for 1 study (4%), moderate for 18 (72%), and low for 6 (24%). Most studies were conducted in the United Kingdom (*k*=6), followed by Finland (*k*=5), Canada (*k*=4), Italy (*k*=3), and Tunisia (*k*=2). Single studies came from Belgium, France, Norway, Brazil, and the United States.

**Table 1:** Characteristics of studies included in the systematic review and meta-analysis on adverse occupational outcomes (AOO) among workers with occupational asthma (OA). [admin=administrative; CS=cross-sectional; CSL=chronic sick leave; D=disability; ER=early retirement; L=longitudinal; SIC=specific inhalation challenge; U=unemployed.]

Study	Country	Design	Follow-up (years)	N	Age, mean (SD)	Male (%)	Diagnosis method	Outcomes reported	Events / N	Preval-ence (%)	Quality ^a^
Burge, 1982 (19)	UK	L	2.4	28	50.0 (6.1)	7	SIC-confirmed	U	13 / 28	46	Moderate
Gannon et al, 1993 (20)	UK	L	3.0	112	48.0 (14.8)	74	Mixed/clinical	CSL, ER, U	39 / 112	35	Moderate
Marabini et al, 1993 (21)	Canada	L	5.5	128	46.9 (1.8)	100	SIC-confirmed	ER, U	53 / 128	41	Moderate
Cannon et al, 1995 (13)	UK	CS	—	87	40.0 (—)	60	Mixed/clinical	— b	34 / 87	39	Moderate
Ameille et al, 1997 (9)	France	L	3.1	202	38.0 (—)	75	Mixed/clinical	CSL, U	65 / 202	32	Moderate
Kujala et al, 1997 (22)	Finland	CS	—	32	41.2 (9.8)	0	Mixed/clinical	ER, U	4 / 32	12	Moderate
Gassert et al, 1998 (23)	USA	L	2.6	55	41.1 (9.8)	29	Mixed/clinical	U	38 / 55	69	Low
Ross et al, 1998 (14)	UK	L	4.0	701	40.9 (12.4)	71	Mixed/clinical	— b	285 / 701	41	Low
Moscato et al,1999 (24)	Italy	L	1.0	25	35.5 (14.5)	72	SIC-confirmed	U	1 / 25	4	Moderate
Larbanois et al, 2002 (25)	Belgium	L	3.8	84	38.0 (11.3)	62	SIC-confirmed	CSL, U	31 / 84	37	Moderate
Piirilä et al, 2005 (26)	Finland	L	10.4	213	50.5 (11.4)	78	Mixed/clinical	— b	120 / 213	56	Moderate
Leira et al, 2005 (15)	Norway	CS	—	723	48.0 (—)	76	Registry/admin	CSL, D, U	343 / 723	47	Moderate
Brant et al, 2006 (27)	UK	L	3.1	35	42.0 (2.0)	77	Mixed/clinical	U	15 / 35	43	High
Hannu et al, 2007 (28)	Finland	L	0.5	31	44.7 (—)	100	SIC-confirmed	D, U	21 / 31	68	Moderate
Malo et al, 2008 (29)	Canada	CS	—	94	— (—)	72	Registry/admin	U	36 / 94	38	Moderate
Chatti et al, 2011 (30)	Tunisia	CS	—	219	40.0 (8.2)	32	Registry/admin	U	97 / 219	44	Moderate
Talini et al, 2012 (31)	Italy	L	2.6	41	39.6 (11.9)	80	SIC-confirmed	U	9 / 41	22	Moderate
Moullec et al, 2013 (32)	Canada	L	8.0	50	49.0 (12.0)	58	Mixed/clinical	CSL, U	7 / 50	14	Low
Karvala et al, 2014 (33)	Finland	L	7.8	107	52.7 (8.0)	86	Mixed/clinical	D, U	41 / 107	38	Moderate
Lipszyc et al, 2017 (34)	Canada	CS	—	32	58.7 (9.5)	64	Mixed/clinical	U	7 / 32	22	Low
Mezni et al, 2018 (35)	Tunisia	CS	—	127	40.5 (8.0)	41	Registry/admin	U	89 / 127	70	Moderate
Feary et al, 2020 (36)	UK	CS	—	71	— (—)	69	Mixed/clinical	U	17 / 71	24	Moderate
Mason et al, 2023 (37)	Italy	L	10.6	57	39.7 (9.7)	75	SIC-confirmed	U	5 / 57	9	Low
Roio et al, 2023 (10)	Brazil	CS	—	80	49.7 (8.9)	47	Mixed/clinical	D, U	33 / 80	41	Low
Lantto et al, 2024 (6)	Finland	L	9.6	59	53.8 (11.2)	77	Mixed/clinical	CSL, U	18 / 59	31	Moderate

^a^ Quality based on the Joanna Briggs Institute (JBI) “Checklist for Prevalence Studies”. b Only overall adverse occupational outcome derivable (multiple-choice responses, specific outcome not identifiable).

Concerning case ascertainment, 7 studies reported a specific inhalation challenge (SIC) confirmed diagnosis, 4 studies used registry or administrative sources, and 14 reported mixed or clinical approaches. Adverse occupational sub-outcomes reported in the studies include: unemployment (*k*=22), chronic sick leave (*k*=6), disability (*k*=4), and early retirement (*k*=3). Only overall AOO was reported in three studies (13–15); this is indicated in table 1 with missing data (“—”) in the column of reported outcomes.

The included studies cover a wide range of occupational groups, including healthcare personnel, painters, welders, laboratory and research workers, as well as employees in the lumber, detergent, textile, and manufacturing industries. Exposure patterns are accordingly diverse, spanning high- and low-molecular-weight agents, dusts, isocyanates, and other sensitizers. Occupations such as bakery work are represented within these mixed cohorts, but are not the exclusive focus of any single study.

### Prevalence of adverse occupational outcomes

**Figure 2:**
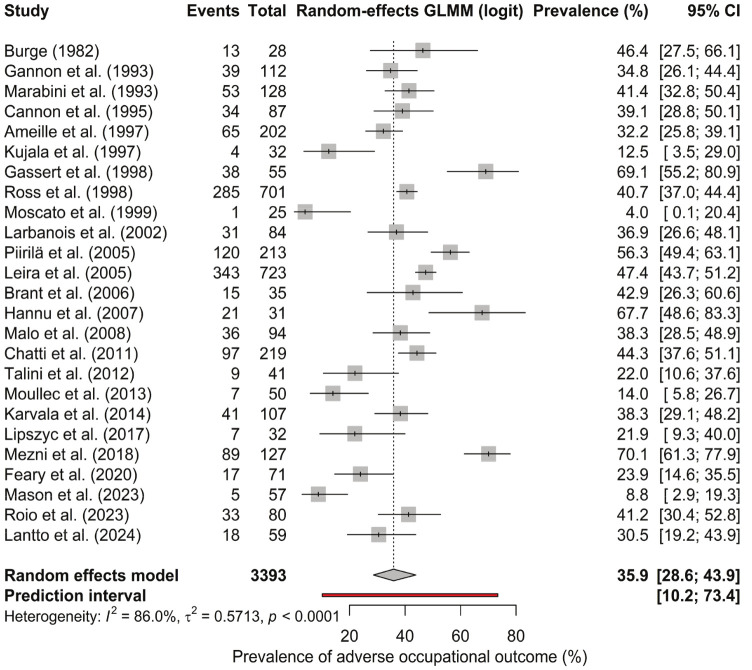
Pooled prevalence of adverse occupational outcome (AOO) in the main analysis. Squares show study estimates (95% confidence intervals [CI]). The diamond represents the pooled estimate from a random-effects GLMM (logit model, maximum-likelihood τ^2^, Hartung–Knapp CI), back-transformed to the proportion scale. The horizontal bar indicates the 95% prediction interval. “Events” refers to the number of observed AOO cases. “Total” refers to the total number of participants assessed for employment status.

Random-effects GLMM with logit transformation presented in figure 2 showed a pooled prevalence of AOO (unemployment, chronic sick leave, disability, or early retirement) of 35.9% (95% CI 28.6–43.9) across 25 studies. Between-study heterogeneity was high (*I*^2^=86.0%, τ^2^=0.571; *Q*=171.11, degrees of freedom=24, P<0.001). The 95% PI was 10.2–73.4%, indicating substantial between-study heterogeneity. Supplementary figure S1 shows country-level pooled prevalence estimates (*k* ≥2), which illustrate marked geographic variation and uneven coverage across regions. Many areas, particularly outside Europe and North America, lacked eligible studies entirely.

The contour-enhanced funnel plot (supplementary figure S2A) suggested asymmetry, and Egger’s regression yielded a borderline result for small-study effects (*t*=-2.06, degrees of freedom=23, P=0.0509). The Baujat plot (supplementary figure S2B) identified a few studies with larger contributions to heterogeneity (*Q*) but uniformly low influence on the pooled effect. As bias-sensitivity checks, limit meta-analysis produced a pooled estimate close to the primary GLMM [37.0% (95% CI 29.4–45.2)] whereas trim-and-fill imputed *k*_0_=7 studies and yielded a higher, exploratory estimate of 44.9% (35.3–54.9%). Leave-one-out refits showed that removing any single study shifted the pooled prevalence by ≤1.7 percentage points, see supplementary figures S3–S4.

### Stratified adverse occupational outcomes

Because our measure of AOO aggregated distinct endpoints, we ran subgroup random-effects GLMM by outcome (see supplementary figure S5). In total, 22 studies contributed 35 outcome-specific data points. As mentioned, 3 studies provided only aggregated AOO data (overlapping subcategories >100%) and were therefore not included in the outcome-specific analyses. Pooled prevalences were: (i) unemployment (*k*=22): 21.3% (95% CI 13.4–32.1), *I*^2^=94.4%; (ii) chronic sick leave (*k*=6): 12.5% (95% CI 5.6–25.8), *I*^2^=94.9%; (iii) disability (*k*=4): 24.1% (95% CI 14.9–36.6), *I*^2^=79.5%; (iv) early retirement (*k*=3): 6.7% (95% CI 0.8–38.3), *I*^2^=79.1%.

A formal test for subgroup differences across the four strata indicated significant heterogeneity between outcomes (*Q*=10.51; degrees of freedom=3; P=0.015). Outcome-specific funnel plots (see S6) suggested asymmetry for unemployment and chronic sick leave, whereas disability and early retirement showed limited interpretability due to the small number of contributing studies. Baujat plots highlighted that a few large studies contributed disproportionately to between-study heterogeneity in the unemployment and chronic-sick-leave strata. Overall, disability appeared as the most frequent specific adverse outcome, while early retirement was least common.

### Analysis of specific inhalation challenge-confirmed cases

**Figure 3:**
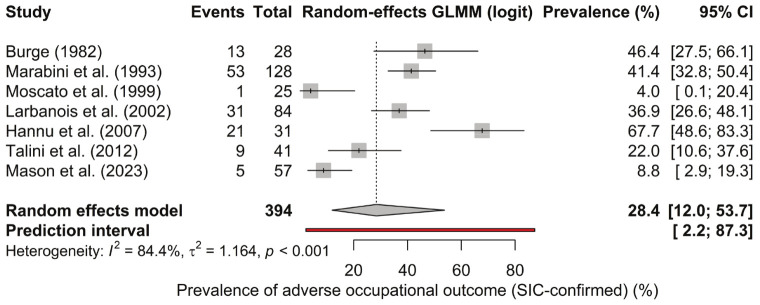
Pooled prevalence of adverse occupational outcome (AOO) restricted to studies with specific inhalation challenge (SIC)-confirmed diagnosis. Squares show study estimates (95% Confidence Intervals [CI]). The diamond represents the pooled estimate from a random-effects GLMM (logit model, maximum-likelihood τ^2^, Hartung–Knapp CI), back-transformed to the proportion scale. The horizontal bar indicates the 95% prediction interval.

To address potential heterogeneity related to diagnostic precision, we performed a sensitivity analysis restricted to studies utilizing SIC as the diagnostic gold standard. Random-effects GLMM with logit transformation as presented in figure 3 showed a pooled prevalence of AOO of 28.4% (95% CI 12.0–53.7) across 7 studies (N=394). Between-study heterogeneity was high (*I*^2^=84.4%; P<0.001). The contour-enhanced funnel plot (supplementary figure S7A) suggested asymmetry and the Baujat plot (supplementary figure S7B) identified specific studies contributing to heterogeneity, though their influence on the overall pooled effect remained moderate. Leave-one-out refits (supplementary figure S8) showed that removing any single study shifted the pooled prevalence approximately 23–35%, indicating a higher sensitivity to individual study results compared to the main analysis. As bias-sensitivity checks, limit meta-analysis and trim-and-fill (supplementary figure S9) yielded higher adjusted estimates, suggesting that the observed prevalence of 28.4% may be conservative. Formal testing for funnel plot asymmetry (Egger’s regression) was not performed due to the insufficient number of studies (*k* <10).

### Subgroup analyses

Table 2 summarizes pooled prevalences and heterogeneity within subgroups, with between-group P-values from *Q*-tests. Again, overall prevalence of AOO was 35.9%. Most splits showed no clear differences (publication year, sex, age, follow-up, study quality; all P>0.05). Prevalence was higher in registry/compensation settings (50.1%, 31.8–68.4; *I*^2^=89%) than clinic-based studies (32.0%, 23.6–41.7; *I*^2^=85%; P=0.015). Regional patterns were different (P=0.027) with a prevalence of 19.2% (7.1–42.2) for continental Europe, 28.9% (13.4–51.7) for Canada, 37.8% (31.2–44.8) for the UK, 41.5% (24.4–61.0) for Fennoscandia, and 52.2% (22.3–80.7) for South America and Africa. Larger studies (>80 participants) reported higher prevalence than smaller ones (≤80 participants): 43.4% versus 27.9% (P=0.033). Several clinical subgroups were associated with different prevalences: baseline FEV_1_ ≤85% predicted (38.2%) versus >85% (13.8%; P=0.008); SIC-confirmed diagnosis (28.4%) versus mixed/clinical (35.1%) and registry/administrative (50.1%; borderline P=0.061); longer latency from exposure onset to symptoms (>7.1 years: 35.7% versus ≤7.1 years: 15.7%; P=0.015). Subgroups based on FVC at baseline, ΔFEV_1_, and symptom duration to diagnosis were imprecise and not significant, largely because these comparisons included <5 studies and thus provided limited statistical power.

**Table 2:** Pooled prevalence of adverse occupational outcome by study-level subgroups (random-effects GLMM on logit scale, ML τ^2^, Hartung–Knapp confidence intervals (CI); back-transformed). P-values derived from between-group Q-tests. [SIC=specific inhalation challenge; FEV_1_=forced expiratory volume in 1 second; FVC=forced vital capacity].

Subgroup	Level	k	N	Prevalence % (95% CI)	I^2^ (%)	P-value
Overall	All studies	25	3393	35.9 (28.6–43.9)	86	—
Publication year	≤2006	13	2425	38.6 (29.3–48.8)	82	0.471
	>2006	12	968	33.3 (22.1–46.8)	89	
Male sex	≤72%	13	1591	34.1 (22.4–48.0)	88	0.650
	>72%	12	1802	37.4 (28.4–47.4)	85	
Age	≤42 years	12	1665	32.8 (20.1–48.8)	89	0.407
	>42 years	11	1563	39.6 (30.5–49.4)	81	
Region	Canada ^a^	4	304	28.9 (13.4–51.7)	78	0.027
	Fennoscandia	6	1165	41.5 (24.4–61.0)	85	
	United Kingdom	6	1034	37.8 (31.2–44.8)	44	
	Continental Europe	5	409	19.2 (7.1–42.2)	79	
	South America & Africa ^a^	3	426	52.2 (22.3–80.7)	92	
Study design	Longitudinal	16	1928	34.9 (25.2–46.0)	85	0.705
	Cross-sectional	9	1465	37.7 (25.9–51.2)	87	
Study size	≤80	13	596	27.9 (16.8–42.5)	86	0.033
	>80	12	2797	43.4 (37.1–49.9)	85	
Follow up	≤3.5 years	8	529	38.0 (21.1–58.4)	85	0.564
	>3.5 years	8	1399	32.2 (20.3–47.0)	87	
Setting	Clinic	19	1494	32.0 (23.6–41.7)	85	0.015
	Registry/compensation ^a^	4	1163	50.1 (31.8–68.4)	89	
Diagnosis	SIC-confirmed	7	394	28.4 (12.0–53.7)	84	0.061
	Mixed/clinical	14	1836	35.1 (27.0–44.2)	84	
	Registry/administrative ^a^	4	1163	50.1 (31.8–68.4)	89	
FEV_1_ T0	≤85% predicted	5	380	38.2 (31.5–45.3)	29	0.008
	>85% predicted ^a^	4	216	13.8 (3.2–43.8)	85	
FVC T0	≤93% predicted ^a^	3	224	35.3 (23.0–49.9)	45	0.592
	>93% predicted ^a^	3	213	28.6 (3.5–81.7)	88	
FEV_1_ Difference (follow-up minus baseline)	≤-1.7% predicted ^a^	3	181	26.1 (1.5–88.9)	75	0.688
	>-1.7% predicted ^a^	2	169	19.4 (0.0–99.8)	91	
Duration of exposure before symptoms onset	≤7.1 years ^a^	2	128	15.7 (0.1–97.5)	79	0.015
	>7.1 years ^a^	2	196	35.7 (7.7–78.7)	0	
Duration of symptoms until diagnosis	≤4.7 years ^a^	3	311	22.9 (2.5–77.8)	69	0.824
	>4.7 years ^a^	3	249	26.0 (4.2–73.9)	86	
Study quality ^b^	Low	6	975	29.6 (12.2–56.0)	90	0.429
	Moderate	18	2383	37.7 (29.9–46.3)	85	

^a^ Stratum with <5 studies. Strata containing <2 studies were excluded from the specific subgroup analyses. ^b^ Quality based on the Joanna Briggs Institute (JBI) Checklist for Prevalence Studies.

### Sensitivity analysis: UK subgroup

Sensitivity analyses focusing on the UK subgroup confirmed the robustness of the findings against potential data overlap. In contrast to the global analysis, heterogeneity within the UK subgroup was notably lower (*I*^2^=44.3%), indicating a more consistent outcome pattern within this national setting. To ensure that the pooled prevalence of 37.8% (95% CI 31.2–44.8) was not driven by the large sentinel registry (14), which potentially overlaps with smaller clinical cohorts included in the analysis (13, 20), we recalculated the estimate excluding these registry data. This exclusion yielded a similar prevalence of 35.6% (95% CI 27.5–44.8%) and further reduced heterogeneity to 41.1% (see supplementary table S3).

## Discussion

This meta-analysis of 25 studies, including 3393 workers with OA, shows that one in three affected workers had an AOO, such as unemployment, chronic sick leave, disability, or early retirement. The prevalence was higher in registry or administrative datasets compared to clinic-based cohorts and increased with greater baseline functional impairment and longer latency between exposure onset and symptom manifestation. These findings suggest that disease severity and diagnostic delay contributed significantly to the heterogeneity observed in the data. However, diagnostic precision alone does not fully explain the variability. Substantial heterogeneity persisted even when restricting the analysis to the diagnostic gold standard of SIC-confirmed cases, indicating that structural differences in social security systems and labor markets were likely the primary drivers. This hypothesis is supported by our sensitivity analysis of the UK subgroup. In contrast to the substantial heterogeneity observed in the global dataset, the UK subgroup, which operates under a unified compensation and healthcare framework, demonstrated notably lower heterogeneity. This finding suggests that a substantial portion of the global variation was attributable to differences in national compensation schemes rather than clinical differences alone. A key observation was the geographic variation in the available literature: while most studies were from Europe and North America, low- and middle-income countries remain underrepresented. Moreover, occupational groups were often studied within mixed cohorts rather than specific industries, which can obscure the identification of occupation-specific risks and limit our ability to draw nuanced conclusions regarding the impact of various workplace exposures.

Our study is one of the first to provide a pooled analysis of the global burden of AOO in OA, offering new insights into how these outcomes manifest across different regions and industries. Previous studies, such as Ameille et al (9), showed that 44% of workers with OA had left their previous job, with significant income reductions especially among those who changed employers. These findings are consistent with those of Henneberger et al (11), who noted that while exposure cessation tended to improve clinical outcomes, it was often accompanied by higher risks of unemployment and income loss . Similarly, Feary et al (36) found that patients removed from causative exposure showed clinical improvement in respiratory symptoms, but experienced poorer employment continuity and income stability. Our study extends these findings by quantifying the burden of AOO across diverse clinical and registry-based study settings and accounting for variability in diagnostic delay, baseline lung function, and exposure duration.

To put the high prevalence of AOO into perspective, we compared our findings with baseline rates in the general population. The pooled unemployment prevalence in our analysis was 21.3%, a figure substantially higher than the general unemployment rate of approximately 4.9% reported for the OECD area in 2024 (38). While older age and the prevalence of manual labor in OA cohorts undoubtedly contribute to higher labor market risk, these factors alone are unlikely to explain a fourfold increase in unemployment. This specific disease burden is further underscored by the observed disability prevalence of 24.1%, which stands in sharp contrast to population-based data. Hakola et al (39), in a large prospective study of public sector employees, reported a disability pension rate of only 2.9% in non-asthmatic controls. Even among employees with persistent general asthma, the rate was approximately 5.8%—substantially lower than the 24.1% observed in our OA cohort. Furthermore, Taponen et al (40) reported a work disability rate of approximately 20% in a general asthma population, illustrating the severe burden of the disease. While our study population included a higher proportion of male manual workers, the disability rate in OA workers was more than eight times that of healthy controls, underscoring the severe impact of OA on work capacity.

OA frequently leads to long-term employment disruption, with significant implications for workers’ financial stability, mental health, and social participation. The findings suggest that effective prevention and management strategies should integrate primary exposure control with ongoing medical surveillance, early specialist referral, and structured return-to-work programs. Close collaboration between clinicians, occupational health services, and regulatory authorities is essential to ensure that workers with OA are not permanently excluded from the labor market. Certain occupations, such as baking and flour processing, remain notably understudied, despite being significant sources of occupational respiratory allergy (41). Industrialization of these sectors and the widespread use of baking improvers and enzyme additives have introduced new allergen sources (eg, fungal α-amylase, glucoamylase, or xylanase), while their precise contribution to the development of OA remains uncertain. At the same time, more industrialized workplaces may offer improved opportunities for technical and individual preventive measures, including better dust control, automation, and structured medical surveillance programs (42). Understanding how affected individuals can safely remain employed, and under what conditions this promotes recovery rather than deterioration, is critical for providing evidence-based guidance and improving occupational health policies. Suojalehto et al (43) emphasize that, even after symptom improvement, workers with sensitizer-induced OA often continue to face significant socioeconomic challenges, particularly in terms of career progression and financial stability. This burden is not limited to sensitizer-induced phenotypes. Recent evidence from Lantto et al (6) suggests that the long-term prognosis of irritant-induced OA is equally severe, or potentially even poorer. This highlights the importance of addressing not only the clinical aspects of OA but also the long-term economic and social impacts that can arise across all phenotypes, even after exposure cessation.

Therefore, future studies should focus on identifying the determinants of job retention and successful recovery among workers with OA. Comparative analyses of those who remain employed versus those who leave the labor market are essential to clarify how factors such as respiratory function, exposure characteristics, psychosocial resources, and socioeconomic context interact to shape long-term outcomes. Harmonized definitions of AOO, standardized denominators, and a shared core outcome set would enhance comparability and reproducibility across studies. Additionally, large, longitudinal, multi-country cohorts and registry linkages that reflect differences in compensation and welfare systems, including data from informal labor markets, are needed to develop globally relevant and equitable guidance. Further research is particularly needed to investigate how specific occupational exposures in underrepresented industries contribute to long-term outcomes in OA.

### Strengths and limitations

This review followed PRISMA recommendations with a preregistered protocol and a comprehensive search in three databases, yielding the largest synthesis to our knowledge of AOO in OA, comprising 25 studies and 3393 participants. Employment endpoints were harmonized to a transparent composite with study-level denominators that excluded ordinary retirement. We applied a random-effects meta-analysis of proportions with logit transformation and reported prediction intervals to reflect between-study variability. The robustness of the findings was evaluated through leave-one-out analyses and formal tests for small-study effects, and the conclusions remained consistent. All analyses are fully reproducible.

Several issues temper the certainty of our estimates. Given the high heterogeneity, which largely reflects inherent contextual differences in healthcare systems, diagnostic procedures, and occupational compositions, the pooled estimate should be interpreted as a summary range rather than a single summary value. Furthermore, our review spans more than four decades (1982–2024). Although subgroup analysis by publication year did not yield statistically significant differences in AOO prevalence, the evolution of labor markets, social security regulations, and diagnostic criteria over this time span likely introduces substantial variability that cannot be fully adjusted for. The reporting of important covariates was limited. Spirometry results and socioeconomic variables were seldom reported, which constrained assessment of effect modification. Because this is a single-arm meta-analysis of proportions without internal comparators, causal inference regarding determinants of employment loss remains limited. Although bias-oriented sensitivity analyses and tests for small-study effects were applied, residual bias cannot be completely excluded. Specifically, while we retained a large registry-based study (14) alongside smaller clinical cohorts (13, 20) to maximize data comprehensiveness, potential partial overlap between these datasets was addressed through sensitivity analyses, which showed the stability of the pooled estimates. Subgroup analyses were based on study-level aggregates, and some strata contained very few studies, which limits their interpretability and means that they should be regarded as exploratory.

### Concluding remarks

More than one-third of workers with OA experience a loss of regular employment through unemployment, chronic sick leave, disability, or early retirement. The findings underline that OA remains a major cause of long-term work disability and socioeconomic burden. Early detection, effective exposure control, and structured return-to-work programs are essential to preserve employability. Future research should harmonize outcome definitions, expand coverage to underrepresented regions and occupations, and identify factors that enable workers to maintain safe and sustainable employment despite disease and exposure.

## Supplementary material

Supplementary material
