# Supplementary material for: Adverse occupational outcome among workers with occupational asthma: A systematic review and meta-analysis of influencing factors
Source: Scand J Work Environ Health. 2026 Jun 26;52(4):349–59. doi: 10.5271/sjweh.4282 (PMC13333171; doi:10.5271/sjweh.4282)
Supplement: Supplementary material [file SJWEH-52-349-S001.pdf]

Adverse occupational outcome among workers with occupational asthma: A systematic review and meta-analysis of influencing factors[1]

by Lukas S Damerau, MS,[2] Matthias W Helm, Dr, Julia Pieter, Dr, Marcial Velasco Garrido, Dr, Volker Harth, Prof, Dr, Hanno Hoven, Prof, Dr, Alexandra M Preisser, Prof, Dr

1. Supplementary Material
2. Correspondence to: Lukas S Damerau, Institute for Occupational and Maritime Medicine (ZfAM), Medical Center Hamburg-Eppendorf (UKE), Seewartenstraße 10, 20459 Hamburg, Germany. [E-mail: l.damerau@uke.de]

Supplementary Item 1: PRISMA 2020 Checklist

Section and Topic	Item #	Checklist item	Location where item is reported
**TITLE**			
Title	1	Identify the report as a systematic review.	Page 1, Title
**ABSTRACT**			
Abstract	2	See the PRISMA 2020 for Abstracts checklist.	Page 1, Abstract
**INTRODUCTION**			
Rationale	3	Describe the rationale for the review in the context of existing knowledge.	Pages 1–2, Introduction
Objectives	4	Provide an explicit statement of the objective(s) or question(s) the review addresses.	Page 2, Introduction
**METHODS**			
Eligibility criteria	5	Specify the inclusion and exclusion criteria for the review and how studies were grouped for the syntheses.	Page 2, Methods (Search strategy and study selection)
Information sources	6	Specify all databases, registers, websites, organisations, reference lists and other sources searched or consulted to identify studies. Specify the date when each source was last searched or consulted.	Page 2, Methods (Search strategy and study selection)
Search strategy	7	Present the full search strategies for all databases, registers and websites, including any filters and limits used.	Page 2, Methods (Search strategy) &

Section and Topic	Item #	Checklist item	Location where item is reported
			Supplementary Table S1
Selection process	8	Specify the methods used to decide whether a study met the inclusion criteria of the review, including how many reviewers screened each record and each report retrieved, whether they worked independently, and if applicable, details of automation tools used in the process.	Page 2, Methods (Search strategy and study selection)
Data collection process	9	Specify the methods used to collect data from reports, including how many reviewers collected data from each report, whether they worked independently, any processes for obtaining or confirming data from study investigators, and if applicable, details of automation tools used in the process.	Page 2, Methods (Data extraction and quality assessment)
Data items	10a	List and define all outcomes for which data were sought. Specify whether all results that were compatible with each outcome domain in each study were sought (e.g. for all measures, time points, analyses), and if not, the methods used to decide which results to collect.	Page 3, Methods (Adverse occupational outcome)
	10b	List and define all other variables for which data were sought (e.g. participant and intervention characteristics, funding sources). Describe any assumptions made about any missing or unclear information.	Page 2, Methods (Data extraction and quality assessment)
Study risk of bias assessment	11	Specify the methods used to assess risk of bias in the included studies, including details of the tool(s) used, how many reviewers assessed each study and whether they worked independently, and if applicable, details of automation tools used in the process.	Page 2, Methods (Data extraction and quality assessment)
Effect measures	12	Specify for each outcome the effect measure(s) (e.g. risk ratio, mean difference) used in the synthesis or presentation of results.	Page 3, Methods (Data synthesis and statistical analysis)
Synthesis methods	13a	Describe the processes used to decide which studies were eligible for each synthesis (e.g. tabulating the study intervention characteristics	Page 2, Methods (Search

Section and Topic	Item #	Checklist item	Location where item is reported
		and comparing against the planned groups for each synthesis (item #5)).	strategy and study selection)
	13b	Describe any methods required to prepare the data for presentation or synthesis, such as handling of missing summary statistics, or data conversions.	Page 3, Methods (Data transformation and harmonization)
	13c	Describe any methods used to tabulate or visually display results of individual studies and syntheses.	Page 3, Methods (Data synthesis) & Pages 4–6, Results
	13d	Describe any methods used to synthesize results and provide a rationale for the choice(s). If meta-analysis was performed, describe the model(s), method(s) to identify the presence and extent of statistical heterogeneity, and software package(s) used.	Page 3, Methods (Data synthesis and statistical analysis)
	13e	Describe any methods used to explore possible causes of heterogeneity among study results (e.g. subgroup analysis, meta-regression).	Page 3, Methods (Data synthesis and statistical analysis)
	13f	Describe any sensitivity analyses conducted to assess robustness of the synthesized results.	Page 3, Methods (Data synthesis and statistical analysis)
Reporting bias assessment	14	Describe any methods used to assess risk of bias due to missing results in a synthesis (arising from reporting biases).	Page 3, Methods (Data synthesis and statistical analysis)
Certainty assessment	15	Describe any methods used to assess certainty (or confidence) in the body of evidence for an outcome.	n/a
**RESULTS**			
	16a	Describe the results of the search and selection process, from the number of records identified in	Pages 3–4, Results (Study

Section and Topic	Item #	Checklist item	Location where item is reported
Study selection		the search to the number of studies included in the review, ideally using a flow diagram.	selection) & Figure 1
	16b	Cite studies that might appear to meet the inclusion criteria, but which were excluded, and explain why they were excluded.	Pages 3–4, Results (Study selection) & Supplementary Table S2
Study characteristics	17	Cite each included study and present its characteristics.	Pages 4–5, Results (Characteristics of included studies) & Table 1
Risk of bias in studies	18	Present assessments of risk of bias for each included study.	Page 4, Results (Characteristics of included studies) & Table 1
Results of individual studies	19	For all outcomes, present, for each study: (a) summary statistics for each group (where appropriate) and (b) an effect estimate and its precision (e.g. confidence/credible interval), ideally using structured tables or plots.	Pages 5–6, Results (Table 1, Figure 2, Figure 3)
Results of syntheses	20a	For each synthesis, briefly summarise the characteristics and risk of bias among contributing studies.	Page 4, Results (Prevalence of adverse occupational outcomes)
	20b	Present results of all statistical syntheses conducted. If meta-analysis was done, present for each the summary estimate and its precision (e.g. confidence/credible interval) and measures of statistical heterogeneity. If comparing groups, describe the direction of the effect.	Page 4, Results (Prevalence of adverse occupational outcomes)
	20c	Present results of all investigations of possible causes of heterogeneity among study results.	Pages 6–7, Results (Subgroup analyses) & Table 2

Section and Topic	Item #	Checklist item	Location where item is reported
	20d	Present results of all sensitivity analyses conducted to assess the robustness of the synthesized results.	Pages 6–7, Results (Analysis of SIC-confirmed cases & Sensitivity analysis: UK subgroup)
Reporting biases	21	Present assessments of risk of bias due to missing results (arising from reporting biases) for each synthesis assessed.	Pages 4–6, Results (Prevalence of adverse occupational outcomes)
Certainty of evidence	22	Present assessments of certainty (or confidence) in the body of evidence for each outcome assessed.	n/a
**DISCUSSION**			
Discussion	23a	Provide a general interpretation of the results in the context of other evidence.	Pages 7–9, Discussion
	23b	Discuss any limitations of the evidence included in the review.	Page 8, Discussion
	23c	Discuss any limitations of the review processes used.	Page 9, Discussion (Strengths and limitations)
	23d	Discuss implications of the results for practice, policy, and future research.	Pages 8–9, Discussion (Concluding remarks)
**OTHER INFORMATION**			
Registration and protocol	24a	Provide registration information for the review, including register name and registration number, or state that the review was not registered.	Page 2, Methods (Search strategy and study selection)

Section and Topic	Item #	Checklist item	Location where item is reported
	24b	Indicate where the review protocol can be accessed, or state that a protocol was not prepared.	Page 2, Methods (Search strategy and study selection)
	24c	Describe and explain any amendments to information provided at registration or in the protocol.	Page 2, Methods (Search strategy and study selection)
Support	25	Describe sources of financial or non-financial support for the review, and the role of the funders or sponsors in the review.	Page 9, Funding and competing interests
Competing interests	26	Declare any competing interests of review authors.	Page 9, Funding and competing interests
Availability of data, code and other materials	27	Report which of the following are publicly available and where they can be found: template data collection forms; data extracted from included studies; data used for all analyses; analytic code; any other materials used in the review.	Supplementary Material

Supplementary Item 2: Data and Code Availability

The full extraction table and the R code used for the meta-analysis are openly available in the Zenodo repository at https://doi.org/10.5281/zenodo.19328785.

Supplementary Table S1. Search strategies for each database.

PubMed:

("Asthma, Occupational"[MeSH Terms] OR "occupational asthma" OR "work-related asthma" OR "work-induced asthma" OR "work-exacerbated asthma" OR "work-aggravated asthma" OR "occupational bronchial asthma" OR "workplace asthma" OR "industrial asthma" OR "work asthma" OR "occupational respiratory disease" OR "work-related respiratory disease" OR "occupational lung disease" OR "work-related lung disease" OR "occupational airway disease" OR "occupational pulmonary disease" OR "work-related respiratory" OR "work-related respiratory condition*" OR "occupational bronchitis" OR "irritant-induced asthma") AND

(("Employment"[MeSH Terms] OR "Employment" OR "job loss" OR "job change" OR "quit job" OR "leave job" OR "career change" OR "unemployment" OR "work cessation" OR "job displacement" OR "job separation" OR "work exit" OR "job departure" OR "job discontinuation" OR "workforce reduction" OR "job transition" OR "workplace change" OR "career interruption" OR "job mobility" OR "work interruption" OR "job turnover") OR

("Disability Evaluation"[MeSH Terms] OR "disability" OR "functional impairment" OR "work incapacity" OR "occupational impairment" OR "functional limitation" OR “sickness leave”))

Scopus:

(TITLE-ABS-KEY("asthma, occupational" OR "occupational asthma" OR "work-related asthma" OR "work-induced asthma" OR "work-exacerbated asthma" OR "work-aggravated asthma" OR "occupational bronchial asthma" OR "workplace asthma" OR "industrial asthma" OR "work asthma" OR "occupational respiratory disease" OR "work-related respiratory disease" OR "occupational lung disease" OR "work-related lung disease" OR "occupational airway disease" OR "occupational pulmonary disease" OR "work-related respiratory" OR "work-related respiratory condition*" OR "occupational bronchitis" OR "irritant-induced asthma")) AND

(TITLE-ABS-KEY("employment" OR "job loss" OR "job change" OR "quit job" OR "leave job" OR "career change" OR "unemployment" OR "work cessation" OR "job displacement" OR "job separation" OR "work exit" OR "job departure" OR "job discontinuation" OR "workforce reduction" OR "job transition" OR "workplace change" OR "career interruption" OR "job mobility" OR "work interruption" OR "job turnover" OR

"disability" OR "functional impairment" OR "work incapacity" OR "occupational impairment" OR "functional limitation") OR “sickness leave”)

Web of Science:

(„asthma, occupational“ OR „occupational asthma“ OR „work-related asthma“ OR „work-induced asthma“ OR „work-exacerbated asthma“ OR „work-aggravated asthma“ OR „occupational bronchial asthma“ OR „workplace asthma“ OR „industrial asthma“ OR „work asthma“ OR „occupational respiratory disease“ OR „work-related respiratory disease“ OR „occupational lung disease“ OR „work-related lung disease“ OR „occupational airway disease“ OR „occupational pulmonary disease“ OR „work-related respiratory“ OR „work-related respiratory condition*“ OR „occupational bronchitis“ OR „irritant-induced asthma“) AND

(„employment“ OR „job loss“ OR „job change“ OR „quit job“ OR „leave job“ OR „career change“ OR „unemployment“ OR „work cessation“ OR „job displacement“ OR „job separation“ OR „work exit“ OR „job departure“ OR „job discontinuation“ OR „workforce reduction“ OR „job transition“ OR „workplace change“ OR „career interruption“ OR „job mobility“ OR „work interruption“ OR „job turnover“ OR

„disability“ OR „functional impairment“ OR „work incapacity“ OR „occupational impairment“ OR „functional limitation“ OR „sickness leave“)

Supplementary Table S2. Studies excluded after full-text screening with reasons for exclusion.

Study	Reason for exclusion
Chan-Yeung, M., S. Lam, and S. Koener, Clinical features and natural history of occupational asthma due to western red cedar (Thuja plicata). The American Journal of Medicine, 1982. 72(3): p. 411-415.	unclear occupational outcome
Deprez, R.D., C. Oliver, and W. Halteman, Variations in Respiratory-Disease Morbidity among Pulp and Paper-Mill Town Residents. Journal of Occupational and Environmental Medicine, 1986. 28(7): p. 486-491.	not occupational Asthma
Chanyeung, M., L. Maclean, and P.L. Paggiaro, Follow-up-Study of 232 Patients with Occupational Asthma Caused by Western Red Cedar (Thuja-Plicata). Journal of Allergy and Clinical Immunology, 1987. 79(5): p. 792-796.	unclear occupational outcome
Mapp, C.E., et al., Persistent Asthma Due to Isocyanates - a Follow-up-Study of Subjects with Occupational Asthma Due to Toluene Diisocyanate (Tdi). American Review of Respiratory Disease, 1988. 137(6): p. 1326-1329.	unclear occupational outcome
Venables, K.M., A.G. Davison, and A.J.N. Taylor, Consequences of Occupational Asthma. Respiratory Medicine, 1989. 83(5): p. 437-440.	overlapping study populations
Wang, J.D., et al., Occupational Asthma Due to Toluene Diisocyanate among Velcro-Like Tape Manufacturers. American Journal of Industrial Medicine, 1988. 14(1): p. 73-78.	< 20 cases of OA
Normand, J.C., et al., Asthma from silk in textile mills. Evolution and socio-professional consequences. Concerning 5 cases. Archives des Maladies Professionnelles de Medecine du Travail et de Securite Sociale, 1990. 51(1): p. 17-20.	< 20 cases of OA
Baker, D.B., et al., Cross-Sectional Study of Platinum Salts Sensitization among Precious Metals Refinery Workers. American Journal of Industrial Medicine, 1990. 18(6): p. 653-664.	no calculations possible
Lagier, F., A. Cartier, and J.L. Malo, Medico-legal statistics on occupational asthma in Quebec between 1989 and 1988. Revue des Maladies Respiratoires, 1990. 7(4): p. 337-341.	no full text
Blanc, P.D., et al., Work Disability among Adults with Asthma. Chest, 1993. 104(5): p. 1371-1377.	not occupational Asthma
Gannon, P.F. and P.S. Burge, The SHIELD scheme in the West Midlands Region, United Kingdom. Midland Thoracic Society Research Group. Br J Ind Med, 1993. 50(9): p. 791-6.	overlapping study populations
Malo, J.L., et al., The Quebec System of Compensation for Occupational Asthma - Description, Effectiveness and Cost. Revue Des Maladies Respiratoires, 1993. 10(4): p. 313-323.	no full text
Pisati, G., A. Baruffini, and S. Zedda, Toluene Diisocyanate Induced Asthma - Outcome According to Persistence or Cessation of Exposure. British Journal of Industrial Medicine, 1993. 50(1): p. 60-64.	unclear occupational outcome

Malo, J.L., et al., Prevalence of Occupational Asthma among Workers Exposed to Eastern White Cedar. American Journal of Respiratory and Critical Care Medicine, 1994. 150(6): p. 1697-1701.	< 20 cases of OA
Marabini, A., et al., Response to bronchoprovocation test and outcome of occupational asthma. A follow-up study of subjects with TDI asthma. Medicina del Lavoro, 1994. 85(2): p. 134-141.	no full text
Pisati, G. and S. Zedda, Outcome of Occupational Asthma Due to Cobalt Hypersensitivity. Science of the Total Environment, 1994. 150(1-3): p. 167-171.	< 20 cases of OA
Axon, E.J., J.R. Beach, and P.S. Burge, A comparison of some of the characteristics of patients with occupational and non-occupational asthma. Occupational Medicine, 1995. 45(2): p. 109-111.	overlapping study populations
Baur, X., et al., A Clinical and Immunological Study on 92 Workers Occupationally Exposed to Anhydrides. International Archives of Occupational and Environmental Health, 1995. 67(6): p. 395-403.	not occupational Asthma
Douglas, J.D.M., et al., Occupational Asthma Caused by Automated Salmon Processing. Lancet, 1995. 346(8977): p. 737-740.	unclear occupational outcome
Tarlo, S.M., et al., A Workers Compensation Claim Population for Occupational Asthma - Comparison of Subgroups. Chest, 1995. 107(3): p. 634-641.	unclear occupational outcome
Ameille, J., et al., Occupational asthma: A dark social prognosis, an unsuited compensation. Archives des Maladies Professionnelles et de Medecine du Travail, 1996. 57(7): p. 501-507.	no full text
Assoufi, B.K., et al., Outcome of occupational asthma due to platinum salts. Thorax, 1996. 51(SUPPL. 3): p. A41.	no full text
Blanc, P.D., et al., Asthma, employment status, and disability among adults treated by pulmonary and allergy specialists. Chest, 1996. 109(3): p. 688-696.	not occupational Asthma
Blanc, P.D., et al., Asthma-related work disability in Sweden: The impact of workplace exposures. American Journal of Respiratory and Critical Care Medicine, 1999. 160(6): p. 2028-2033.	not occupational Asthma
Brhel, P. and A. Rihova, The fate of persons affected by occupational asthma. Pracovni Lekarstvi, 1999. 51(2): p. 76-81.	unable to translate
Brisman, J. and B. Järvholm, Bakery work, atopy and the incidence of self-reported hay fever and rhinitis. European Respiratory Journal, 1999. 13(3): p. 502-507.	not occupational Asthma
Chan-Yeung, M., et al., Airway inflammation, exhaled nitric oxide, and severity of asthma in patients with western red cedar asthma. American Journal of Respiratory and Critical Care Medicine, 1999. 159(5): p. 1434-1438.	unclear occupational outcome
Chatkin, J.M., et al., The outcome of asthma related to workplace irritant exposures: a comparison of irritant-induced asthma and irritant aggravation of asthma. Chest, 1999. 116(6): p. 1780-5.	< 20 cases of OA

Leino, T., et al., Health reasons for leaving the profession as determined among Finnish hairdressers in 1980-1995. International Archives of Occupational and Environmental Health, 1999. 72(1): p. 56-59.	can't differentiate between Occupational Asthma and Work-Related Asthma/Unclear Occupational Asthma
Merget, R., et al., Outcome of occupational asthma due to platinum salts after transferral to low-exposure areas. International Archives of Occupational and Environmental Health, 1999. 72(1): p. 33-39.	unclear occupational outcome
Pal, T.M., et al., Follow up investigation of workers in synthetic fibre plants with humidifier disease and work related asthma. Occupational and Environmental Medicine, 1999. 56(6): p. 403-410.	< 20 cases of OA
Smith, T.A. and J. Patton, Health surveillance in milling, baking and other food manufacturing operations - five years' experience. Occupational Medicine-Oxford, 1999. 49(3): p. 147-153.	unclear occupational outcome
Sorgdrager, B., et al., Factors affecting FEV1 in workers with potroom asthma after their removal from exposure. International Archives of Occupational and Environmental Health, 2000. 74(1): p. 55-58.	unclear occupational outcome
Harber, P., et al., Frequency of occupational health concerns in general clinics. Journal of Occupational and Environmental Medicine, 2001. 43(11): p. 939-945.	not occupational Asthma
Kor, A.C., et al., Occupational asthma in Singapore. Singapore Medical Journal, 2001. 42(8): p. 373-377.	unclear occupational outcome
Fourgaut, G., et al., Baker's asthma: Clinical study of 27 cases and the need for a decisional algorithm. Revue Francaise D Allergologie Et D Immunologie Clinique, 2002. 42(7): p. 633-639.	< 20 cases of OA
Redlich, C.A., et al., Diisocyanate-exposed auto body shop workers: A one-year follow-up. American Journal of Industrial Medicine, 2002. 42(6): p. 511-518.	< 20 cases of OA
Vandenplas, O., et al., Occupational asthma caused by natural rubber latex: Outcome according to cessation or reduction of exposure. Journal of Allergy and Clinical Immunology, 2002. 109(1): p. 125-130.	overlapping study populations
Acero, S., et al., Occupational asthma from natural rubber latex: Specific inhalation challenge test and evolution. Journal of Investigational Allergology and Clinical Immunology, 2003. 13(3): p. 155-161.	no full text
Bernstein, D.I., et al., Clinical and occupational outcomes in health care workers with natural rubber latex allergy. Annals of Allergy Asthma & Immunology, 2003. 90(2): p. 209-213.	not occupational Asthma
Henneberger, P.K., et al., Work-related reactive airways dysfunction syndrome cases from surveillance in selected US states. Journal of Occupational and Environmental Medicine, 2003. 45(4): p. 360-368.	unclear occupational outcome
Marabini, A., et al., Outcome of occupational asthma in patients with continuous exposure - A 3-year longitudinal study during pharmacologic treatment. Chest, 2003. 124(6): p. 2372-2376.	< 20 cases of OA

Tabar-Purroy, A.I., et al., Carmine (E-120)-induced occupational asthma revisited. Journal of Allergy and Clinical Immunology, 2003. 111(2): p. 415-419.	< 20 cases of OA
Funakoshi, M., et al., Work-relatedness of asthma among outpatients. Japanese Journal of Allergology, 2004. 53(11): p. 1123-1130.	no full text
Janicke, N., et al., Public health relevance of cattle-allergic farmers in Germany. Allergo Journal, 2004. 13(8): p. 515-516.	unclear occupational outcome
Al-Otaibi, S., S.M. Tarlo, and R. House, Quality of life in patients with latex allergy. Occupational Medicine-Oxford, 2005. 55(2): p. 88-92.	<20 cases of OA
Bakirci, N., et al., Predictors of early leaving from the cotton spinning mill environment in newly hired workers. Occupational and Environmental Medicine, 2006. 63(2): p. 126-130.	can't differentiate between Occupational Asthma and Work-Related Asthma/Unclear Occupational Asthma
Howse, D., et al., Gender and snow crab occupational asthma in Newfoundland and Labrador, Canada. Environmental Research, 2006. 101(2): p. 163-174.	unclear occupational outcome
Dimich-Ward, H., et al., Quality of life and employment status of workers with western red cedar asthma. Journal of Occupational and Environmental Medicine, 2007. 49(9): p. 1040-1045.	overlapping study populations
Heutelbeck, A.R.R., et al., German cattle allergy study (CAS):: public health relevance of cattle-allergic farmers. International Archives of Occupational and Environmental Health, 2007. 81(2): p. 201-208.	unclear occupational outcome
Nienhaus, A., et al., Outcome of Occupational Latex Allergy-Work Ability and Quality of Life. Plos One, 2008. 3(10).	not occupational Asthma
Chiry, S., et al., Frequency of work-related respiratory symptoms in workers without asthma. American Journal of Industrial Medicine, 2009. 52(6): p. 447-454.	can't differentiate between Occupational Asthma and Work-Related Asthma/Unclear Occupational Asthma
Power, S., J. Gallagher, and S. Meaney, Quality of life in health care workers with latex allergy. Occupational Medicine-Oxford, 2010. 60(1): p. 62-65.	can't differentiate between Occupational Asthma and Work-Related Asthma/Unclear Occupational Asthma
Broding, H.C., et al., Course of Occupational Asthma Depending on the Duration of Workplace Exposure to Allergens - a Retrospective Cohort Study in Bakers and Farmers. Annals of Agricultural and Environmental Medicine, 2011. 18(1): p. 35-40.	unclear occupational outcome
Kauppi, P., et al., Short-term prognosis of occupational asthma in a Finnish population. Clinical Respiratory Journal, 2011. 5(3): p. 143-149.	< 20 cases of OA
Labrecque, M., et al., Medical surveillance programme for diisocyanate exposure. Occupational and Environmental Medicine, 2011. 68(4): p. 302-307.	unclear occupational outcome
Miedinger, D., et al., Identification of clinically significant psychological distress and psychiatric morbidity by	overlapping study populations

examining quality of life in subjects with occupational asthma. Health and Quality of Life Outcomes, 2011. 9.	
Miedinger, D., et al., Quality-of-Life, Psychological, and Cost Outcomes 2 Years After Diagnosis of Occupational Asthma. Journal of Occupational and Environmental Medicine, 2011. 53(3): p. 231-238.	overlapping study populations
Murgia, N., et al., Risk factors for respiratory work disability in a cohort of pulp mill workers exposed to irritant gases. Bmc Public Health, 2011. 11.	not occupational Asthma
Di Giampaolo, L., et al., The persistence of allergen exposure favors pulmonary function decline in workers with allergic occupational asthma. International Archives of Occupational and Environmental Health, 2012. 85(2): p. 181-188.	unclear occupational outcome
Mazurek, J.M., G.E. Knoeller, and J.E. Moorman, Effect of current depression on the association of work-related asthma with adverse asthma outcomes: A cross-sectional study using the Behavioral Risk Factor Surveillance System. Journal of Affective Disorders, 2012. 136(3): p. 1135-1142.	can't differentiate between Occupational Asthma and Work-Related Asthma/Unclear Occupational Asthma
Mounchetrou, I.N., et al., Predictors of early cessation of dairy farming in the French Doubs province: 12-year follow-up. American Journal of Industrial Medicine, 2012. 55(2): p. 136-142.	not occupational Asthma
Knoeller, G.E., J.M. Mazurek, and J.E. Moorman, Health-related quality of life among adults with work-related asthma in the United States. Quality of Life Research, 2013. 22(4): p. 771-780.	not occupational Asthma
Talini, D., et al., Mild Improvement in Symptoms and Pulmonary Function in a Long-Term Follow-Up of Patients with Toluene Diisocyanate-Induced Asthma. International Archives of Allergy and Immunology, 2013. 161(2): p. 189-194.	unclear occupational outcome
White, G.E., J.M. Mazurek, and J.E. Moorman, Work-related asthma and employment status-38 states and District of Columbia, 2006-2009. Journal of Asthma, 2013. 50(9): p. 954-959.	can't differentiate between Occupational Asthma and Work-Related Asthma/Unclear Occupational Asthma
Karvala, K., et al., Quality of life of patients with asthma related to damp and moldy work environments. Scandinavian Journal of Work Environment & Health, 2013. 39(1): p. 96-105.	overlapping study populations
Diver, S., et al., Employment status and risk of occupational asthma based on job title in a district general severe asthma treatment cohort. European Respiratory Journal, 2014. 44.	publication type
Gui, W., et al., Inception Cohort Study of Workers Exposed to Toluene Diisocyanate at a Polyurethane Foam Factory: Initial One-Year Follow-Up. American Journal of Industrial Medicine, 2014. 57(11): p. 1207-1215.	<20 cases of OA
Moscato, G., et al., OCCUPATION study (OCCUPationl Asthma: a naTIONal based study): A survey on occupational asthma awareness among italian allergists.	unclear occupational outcome

European Annals of Allergy and Clinical Immunology, 2014. 46(1): p. 26-29.	
Munoz, X., et al., Evolution of occupational asthma: Does cessation of exposure really improve prognosis? Respiratory Medicine, 2014. 108(9): p. 1363-1370.	unclear occupational outcome
Dumas, O., et al., Asthma history, job type and job changes among US nurses. Occupational and Environmental Medicine, 2015. 72(7): p. 482-488.	not occupational Asthma
Mehrparvar, A.H., H.E. Sani, and A. Hakimi, Return to work among patients with occupational asthmament. Iran Occupational Health, 2015. 12(4): p. 40-46.	unable to translate
Nemer, M., et al., Lung function and respiratory symptoms among female hairdressers in Palestine: a 5-year prospective study. Bmj Open, 2015. 5(10).	not occupational Asthma
Talini, D., et al., Sputum eosinophilia is a determinant of FEV1 decline in occupational asthma: results of an observational study. Bmj Open, 2015. 5(1).	unclear occupational outcome
Fell, A.K.M., et al., Breath-taking jobs: a case-control study of respiratory work disability by occupation in Norway. Occupational and Environmental Medicine, 2016. 73(9): p. 600-606.	not occupational Asthma
Lavoie, K.L., et al., Prospective Impact of Psychiatric Disorders on Employment Status and Health Care Use in Patients Investigated for Occupational Asthma. Journal of Occupational and Environmental Medicine, 2016. 58(12): p. 1196-1201.	overlapping study populations
Henchi, M.A., et al., The Quality of Life and Professional Future among Tunisian Workers Suffering from Occupational Asthma. Recent Patents on Inflammation & Allergy Drug Discovery, 2017. 11(1): p. 64-70.	unclear occupational outcome
Nesrine, K., et al., Identificated exposures and its consequences on employment in patients with occupational asthma. European Respiratory Journal, 2017. 50.	publication type
Omrane, A., et al., Occupational asthma compensation in the tunisian center: Cross-sectional study over a period of eight years. Pan African Medical Journal, 2017. 26.	unclear occupational outcome
Beyan, A.C., Y. Demiral, and A. Cimrin, Employment status changes of workers after referral to an occupational disease clinic. Journal of Occupational Health, 2018. 60(6): p. 494-501.	can’t differentiate between Occupational Asthma and Work-Related Asthma/Unclear Occupational Asthma
Bradshaw, L., et al., Work aggravated asthma in Great Britain: a cross-sectional postal survey. Primary Health Care Research and Development, 2018. 19(6): p. 561-569.	not occupational Asthma
Taponen, S., et al., Employment status and changes in working career in relation to asthma: a cross-sectional survey. Journal of Occupational Medicine and Toxicology, 2018. 13.	not occupational Asthma
Desalu, O.O., et al., Physical and socioeconomic impact of asthma in Nigeria: Experience of patients attending three tertiary hospitals. Nigerian Journal of Clinical Practice, 2019. 22(6): p. 855-861.	not occupational Asthma

Reilly, M.J., L. Wang, and K.D. Rosenman, The Burden of Work-related Asthma in Michigan, 1988-2018. Annals of the American Thoracic Society, 2020. 17(3): p. 284-292.	can't differentiate between Occupational Asthma and Work-Related Asthma/Unclear Occupational Asthma
Jo, W., et al., Clinical Importance of Work-Exacerbated Asthma: Findings From a Prospective Asthma Cohort in a Highly Industrialized City in Korea. Allergy Asthma & Immunology Research, 2021. 13(2): p. 256-270.	< 20 cases of OA
Hiller, J., A. Greiner, and H. Drexler, Respiratory afflictions during hairdressing jobs: case history and clinical evaluation of a large symptomatic case series. Journal of Occupational Medicine and Toxicology, 2022. 17(1).	< 20 cases of OA
Romero-Mesones, C., et al., Disposition of Work-Related Asthma in a Spanish Asthma Cohort: Comparison of Asthma Severity Between Employed and Retired Workers. Journal of Allergy and Clinical Immunology-in Practice, 2023. 11(11).	can't differentiate between Occupational Asthma and Work-Related Asthma/Unclear Occupational Asthma
Gaddour, A., et al., An epidemiological and evolutionary profile of occupational asthma of Tunisian workers. Revue Des Maladies Respiratoires, 2024. 41(3).	no full text
Suarthana, E., et al., Work-Related Asthma and Its Impact on Quality of Life and Work Productivity. Journal of Allergy and Clinical Immunology-in Practice, 2024. 12(2).	can't differentiate between Occupational Asthma and Work-Related Asthma/Unclear Occupational Asthma

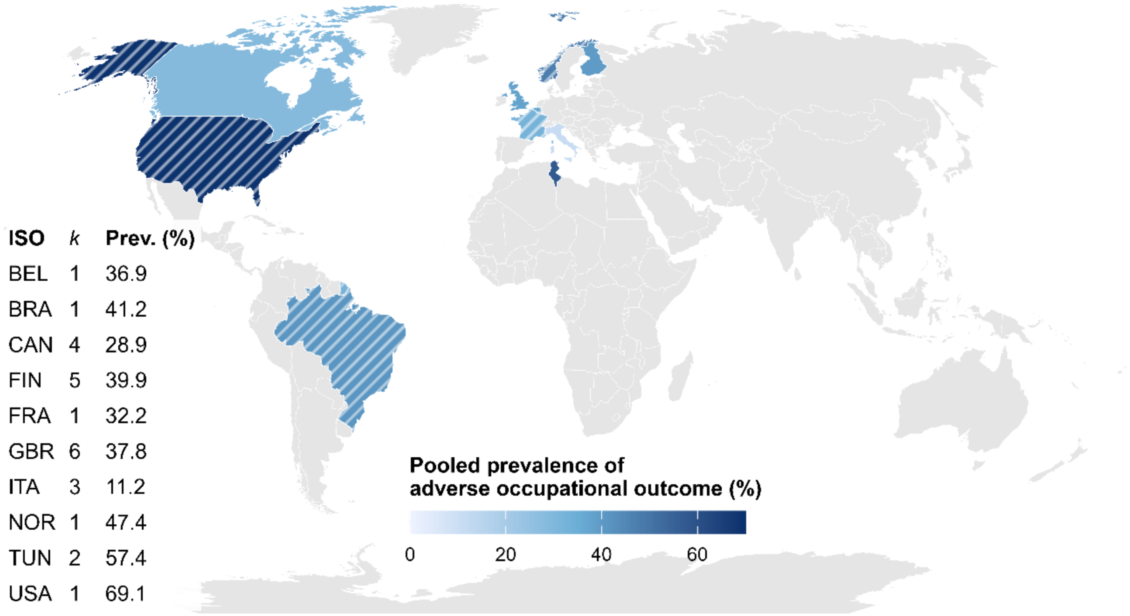

Figure S1. Pooled prevalence (%) by country using the same random-effects GLMM as the main analysis. Filled countries show pooled estimates for $k \geq 2$; countries with $k = 1$ are hatched (single-study only). Labels indicate ISO-3 code and number of studies (k); darker shading indicates higher prevalence. The inset table (left) lists the exact prevalence estimates and k per country.

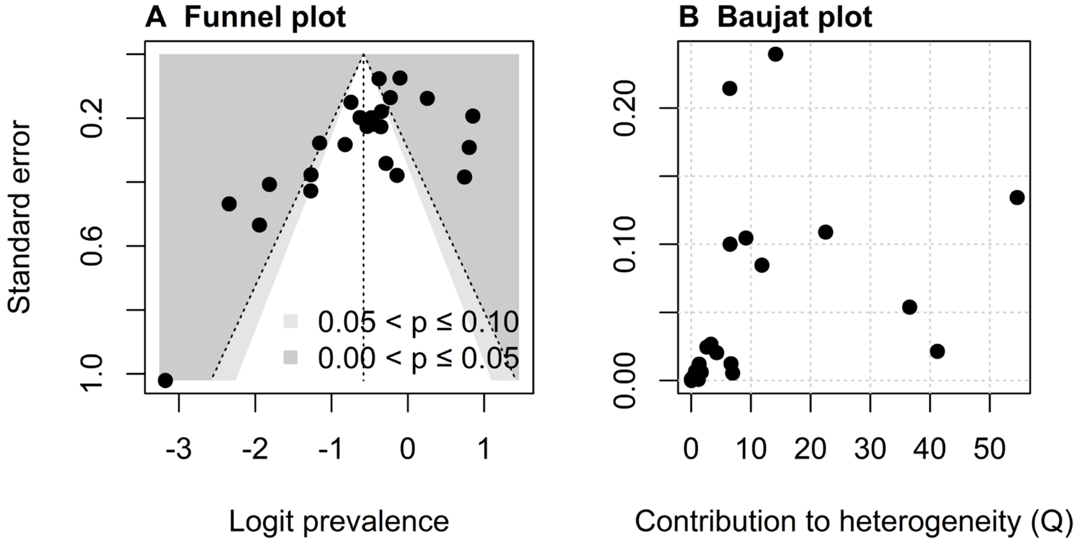

Figure S2. Bias and heterogeneity diagnostics for the main analysis. (A) Contour-enhanced funnel plot centered on the pooled logit prevalence of adverse occupational outcome. The inner, lighter gray band marks 0.05 < *p* ≤ 0.10 and the outer, darker gray band marks 0.00 < *p* ≤ 0.05 relative to the pooled effect. (B) Baujat plot showing each study's contribution to heterogeneity (*Q*, x-axis) versus its influence on the pooled effect (y-axis).

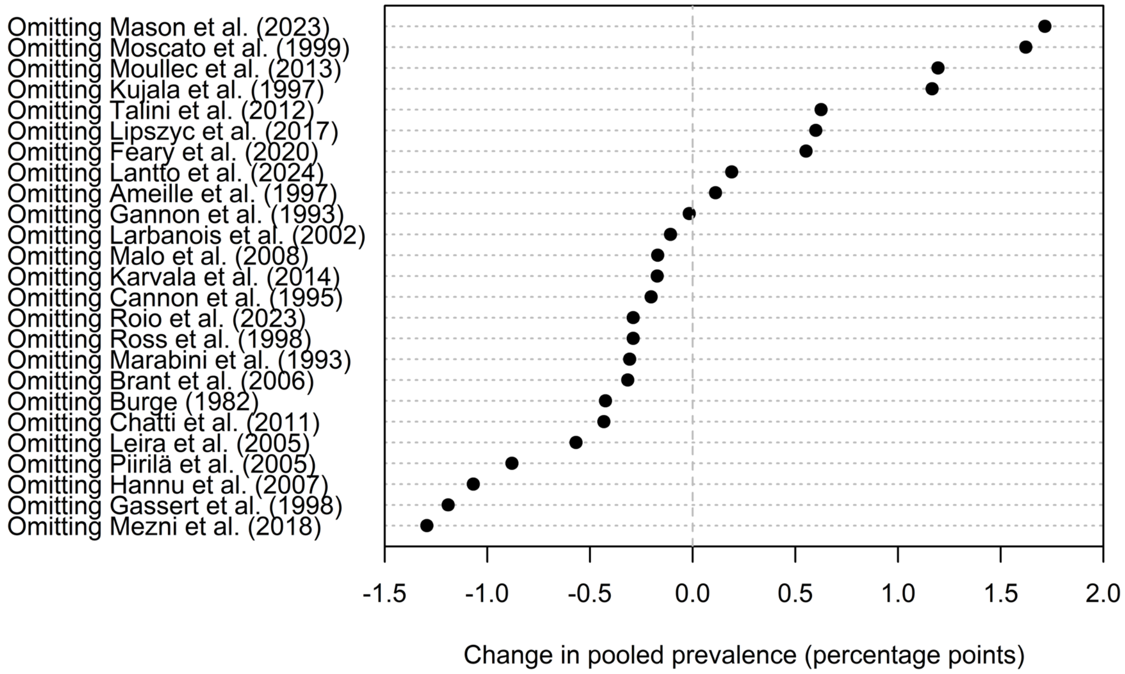

Figure S3. Influence plot showing the change in pooled prevalence of AOO (percentage points) after refitting the random-effects model while omitting one study at a time. Each point represents one excluded study, ordered by influence magnitude. No single study shifted the pooled prevalence by more than ±1.6 percentage points, indicating robust overall estimates.

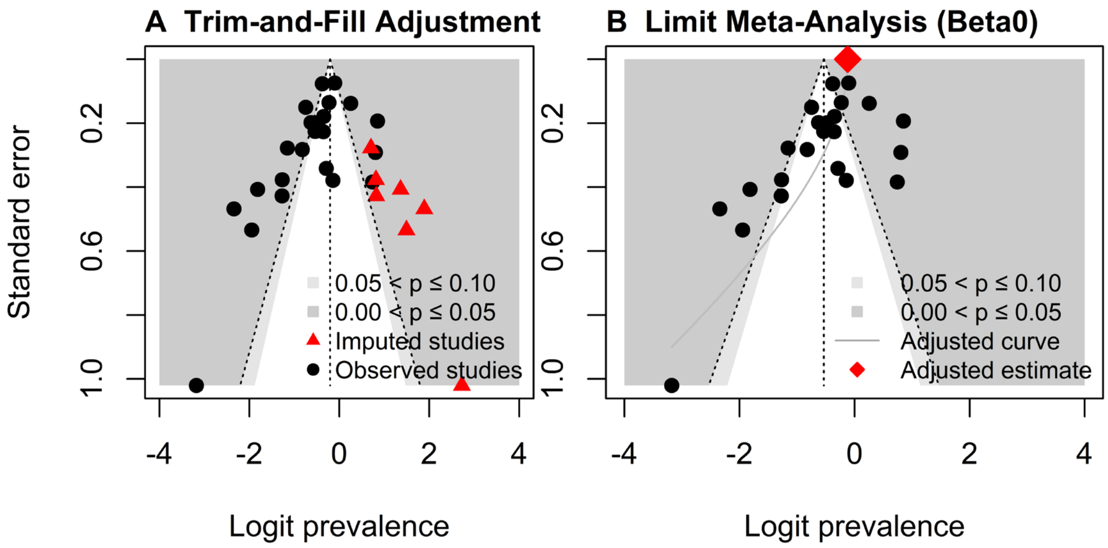

Figure S4. Sensitivity analyses for small-study effects (main analysis) using contour-enhanced funnel plots. Both panels display study precision (standard error) against logit-transformed prevalence. Shaded regions indicate significance contours (white: $p \leq 0.05$; light grey: $0.05 < p \leq 0.10$; dark grey: $p > 0.10$). The vertical dashed line represents the unadjusted pooled random-effects estimate. (A) Duval and Tweedie's trim-and-fill procedure showing observed studies (black circles) and imputed missing studies (red triangles) to correct for asymmetry. (B) Limit meta-analysis (beta0 method) displaying the bias-adjusted regression line (solid grey curve) and the corresponding adjusted pooled estimate (red diamond).

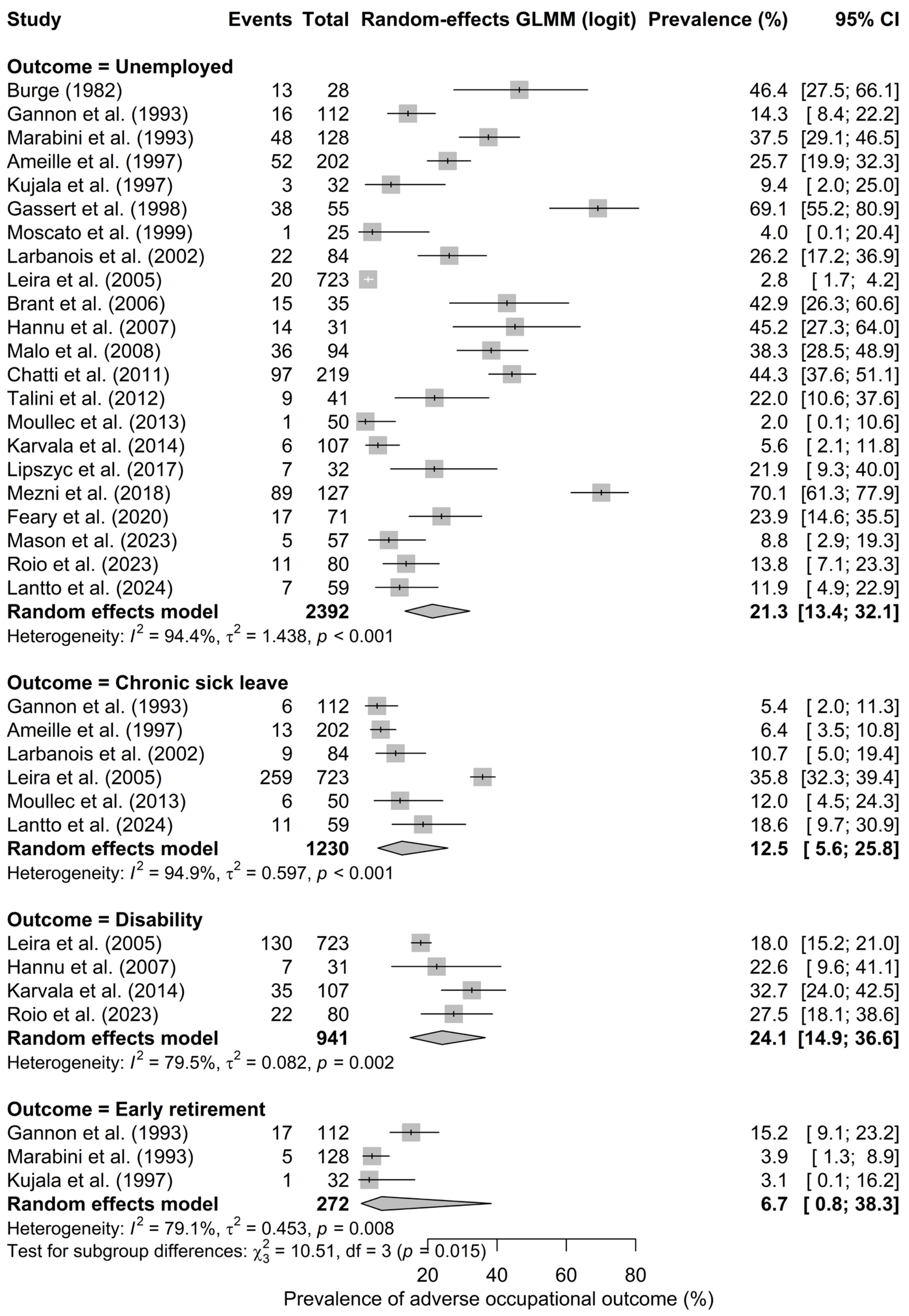

Figure S5. Forest plots showing pooled prevalence estimates of adverse occupational outcomes stratified by sub-outcome (unemployment, chronic sick leave, disability, and early retirement). Squares indicate study-level estimates with 95% confidence intervals (CI), and diamonds represent pooled random-effects generalized linear mixed models (GLMM; logit scale, maximum-likelihood τ^2, Hartung–Knapp CI), back-transformed to proportions. “Events”

denote the number of cases with the specified outcome, and “Total” the number of participants assessed for that outcome.

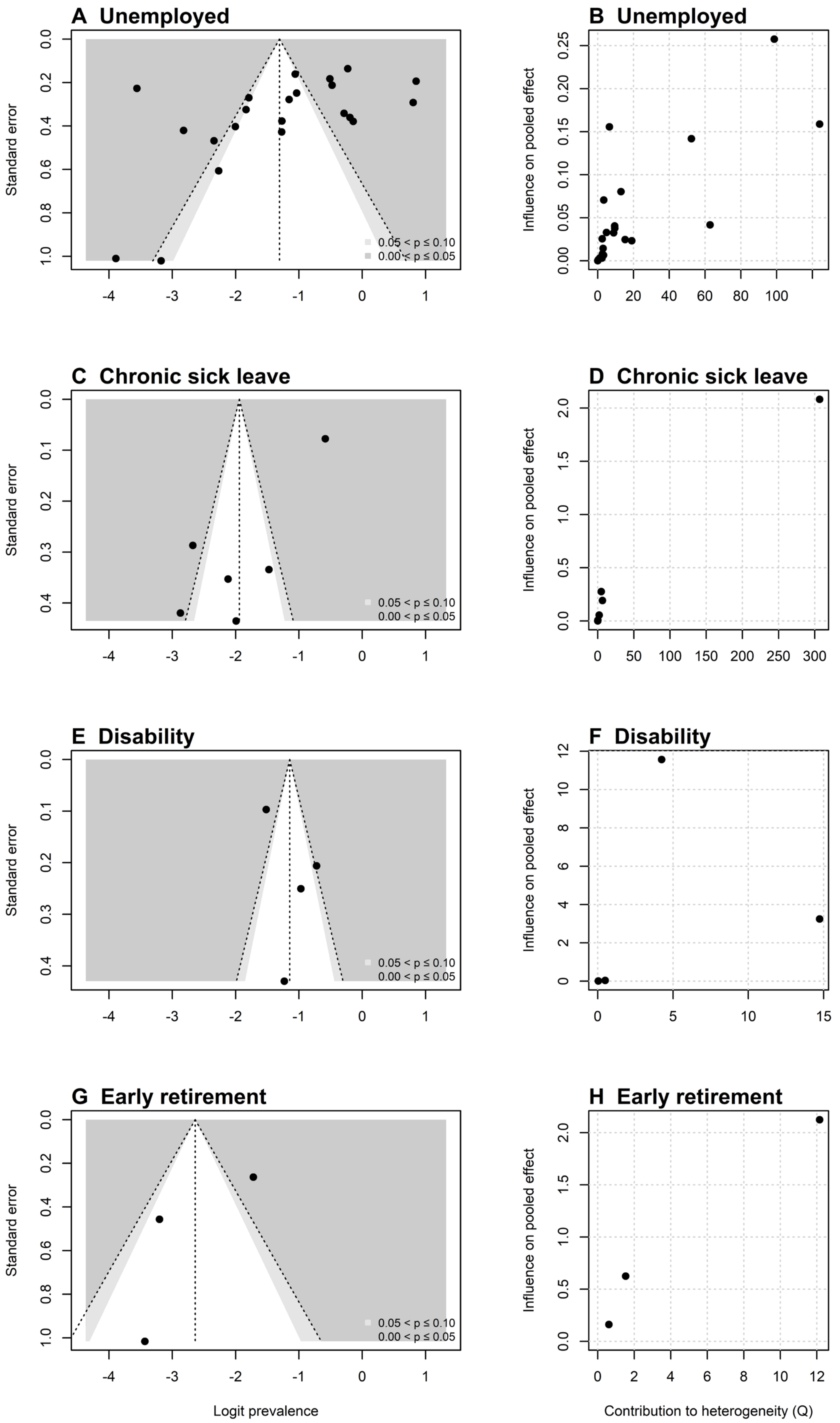

Figure S6. Bias and influence diagnostics by sub-outcome. Contour-enhanced funnel plots (left panels) and Baujat plots (right panels) for individual adverse occupational outcome subtypes: unemployment (A–B), chronic sick leave (C–D), disability (E–F), and early retirement (G–H). Funnel plots show precision (standard error) against logit prevalence; Baujat plots display each study's contribution to heterogeneity (*Q*) and influence on the pooled effect. Across all outcomes, heterogeneity was driven by few small studies, with minimal influence on pooled estimates.

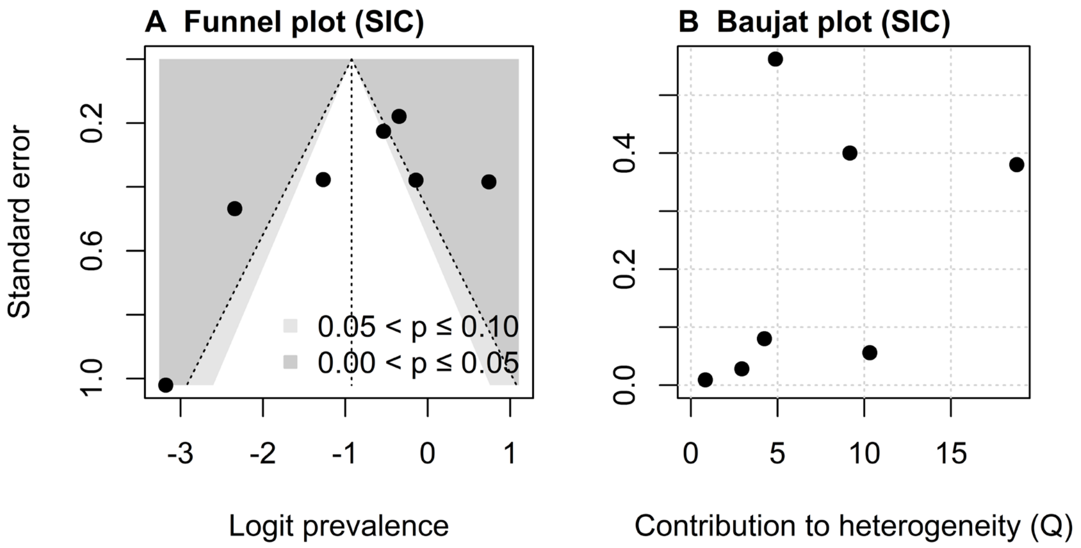

Figure S7. Bias and heterogeneity diagnostics for the specific inhalation challenge (SIC) subgroup. (A) Contour-enhanced funnel plot of study precision (standard error) against logit-transformed prevalence of adverse occupational outcome. Shaded regions indicate significance contours (white: $p \leq 0.05$; light grey: $0.05 < p \leq 0.10$; dark grey: $p > 0.10$). (B) Baujat plot showing each study's contribution to heterogeneity (*Q*, x-axis) versus its influence on the pooled effect (y-axis).

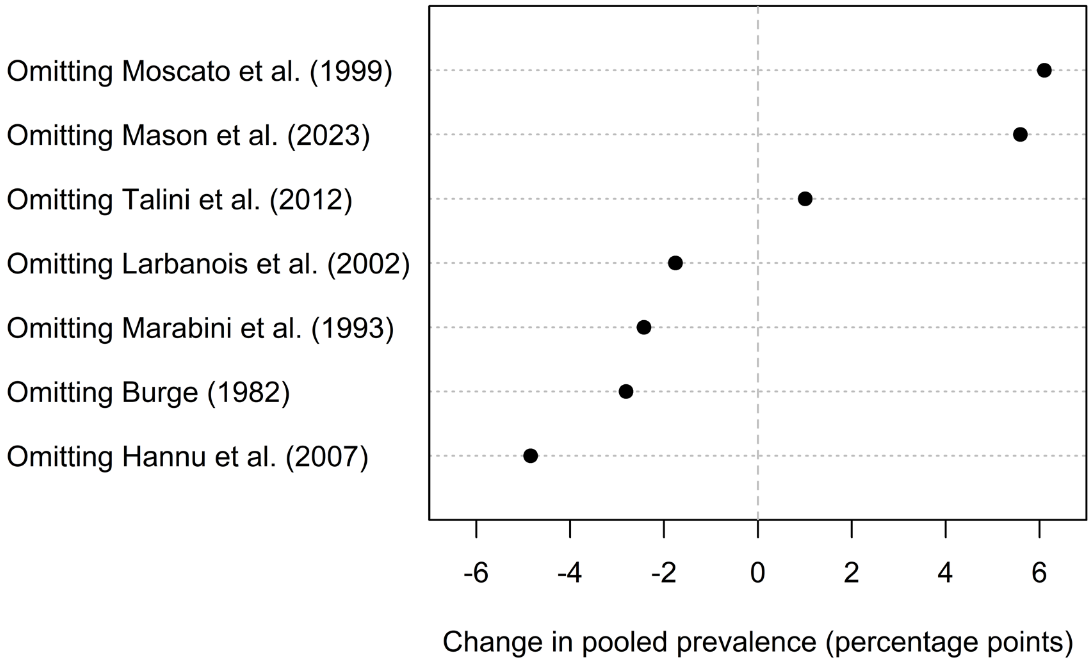

Figure S8. Leave-one-out influence analysis for the specific inhalation challenge (SIC) subgroup. The plot displays the change in pooled prevalence (in percentage points) resulting from re-analyzing the data with each study excluded one at a time. Studies are ordered by the magnitude of their influence on the overall estimate.

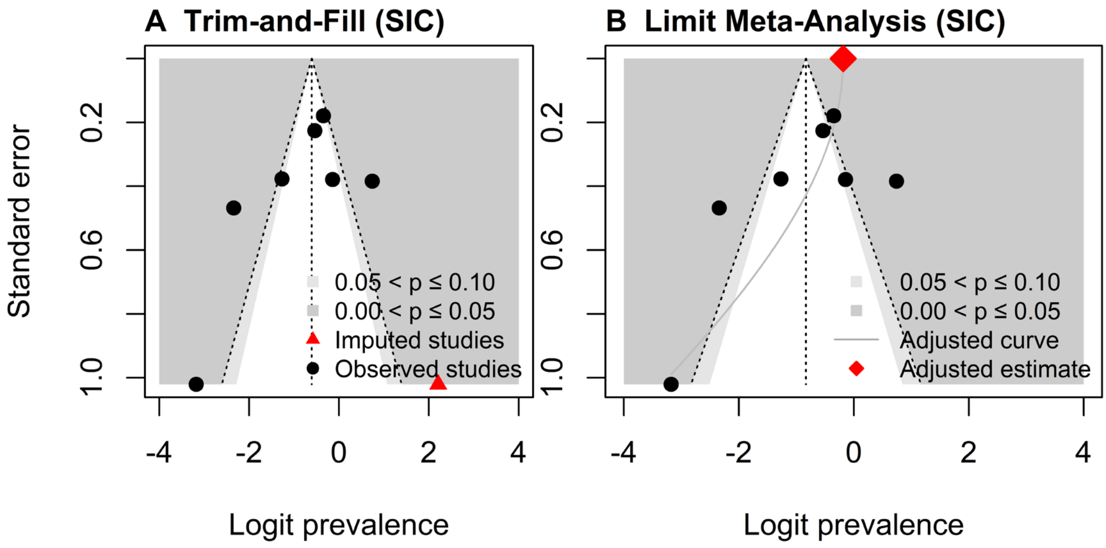

Figure S9. Sensitivity analyses for small-study effects in the specific inhalation challenge (SIC) subgroup using contour-enhanced funnel plots. The vertical dashed line denotes the unadjusted pooled random-effects estimate. (A) Duval and Tweedie's trim-and-fill procedure showing observed studies (black circles) and imputed missing studies (red triangles). (B) Limit meta-analysis (beta0 method) showing the bias-adjusted regression curve (solid grey line) and the corresponding adjusted pooled estimate (red diamond).

Supplementary Table S3. Sensitivity analyses assessing the impact of national registry data (Ross et al).

Scope	Scenario	*k*	*n*	Prevalence % (95 % CI)	I^2 (%)	τ^2
Global Analysis	Primary Model (All 25 studies)	25	3393	35.9 (28.6–43.9)	86.0	0.571
	Sensitivity: Excl. Sentinel Registry (Ross et al.)	24	2692	35.6 (27.9–44.0)	86.4	0.608
UK Subgroup	Primary UK Model	6	1034	37.8 (31.2–44.8)	44.3	0.016
	Sensitivity: Excl. Sentinel Registry (Ross et al.)	5	333	35.6 (27.5–44.8)	41.1	0.021

CI = Confidence Interval
